# Inhibition of breast cancer cell proliferation in repeated and non-repeated treatment with zoledronic acid

**DOI:** 10.1186/1475-2867-12-48

**Published:** 2012-11-22

**Authors:** Toni Ibrahim, Laura Mercatali, Emanuele Sacanna, Anna Tesei, Silvia Carloni, Paola Ulivi, Chiara Liverani, Francesco Fabbri, Michele Zanoni, Wainer Zoli, Dino Amadori

**Affiliations:** 1 Osteoncology Center, IRCCS Istituto Scientifico Romagnolo per lo Studio e la Cura dei Tumori (IRST), via P. Maroncelli 40, 47014, Meldola, FC, Italy; 2 Biosciences Laboratory, IRCCS Istituto Scientifico Romagnolo per lo Studio e la Cura dei Tumori (IRST), via P. Maroncelli 40, 47014, Meldola, FC, Italy

**Keywords:** Bone metastasis, Breast cancer, Cell lines, Zoledronic acid

## Abstract

**Background:**

Zoledronic acid is used to treat bone metastases and has been shown to reduce skeletal-related events and exert antitumor activity. The present *in vitro* study investigates the mechanism of action of Zoledronic Acid on breast cancer cell lines with different hormonal and HER2 patterns. Furthermore, we investigated the efficacy of repeated versus non-repeated treatments.

**Methods:**

The study was performed on 4 breast cancer cell lines (BRC-230, SkBr3, MCF-7 and MDA-MB-231). Non-repeated treatment (single exposure of 168 hrs’ duration) with zoledronic acid was compared with repeated treatment (separate exposures, each of 48 hrs’ duration, for a total of 168 hrs) at different dosages. A dose–response profile was generated using sulforhodamine B assay. Apoptosis was evaluated by TUNEL assay and biomolecular characteristics were analyzed by western blot.

**Results:**

Zoledronic acid produced a dose-dependent inhibition of proliferation in all cell lines. Anti-proliferative activity was enhanced with the repeated treatment, proving to be statistically significant in the triple-negative lines. In these lines repeated treatment showed a cytocidal effect, with apoptotic cell death caused by caspase 3, 8 and 9 activation and decreased RAS and pMAPK expression. Apoptosis was not observed in estrogen receptor-positive line: p21 overexpression suggested a slowing down of cell cycle. A decrease in RAS and pMAPK expression was seen in HER2-overexpressing line after treatment.

**Conclusions:**

The study suggests that zoledronic acid has an antitumor activity in breast cancer cell lines. Its mechanism of action involves the decrease of RAS and RHO, as in osteoclasts. Repeated treatment enhances antitumor activity compared to non-repeated treatment. Repeated treatment has a killing effect on triple-negative lines due to apoptosis activation. Further research is warranted especially in the treatment of triple-negative breast cancer.

## Background

Breast cancer is the most commonly diagnosed cancer in women in developed countries and over 50% of patients have bone involvement at relapse [1-4]. Bone metastasis is a major epidemiological and clinical problem in women with breast cancer, causing pain and other serious complications such as pathologic fracture, spinal cord compression and hypercalcemia with poor quality of life and prognosis [5,6].

The skeleton is characterized by a dynamic balance between osteoclast (induced bone resorption) and osteoblast (stimulated bone formation) bone remodeling, which maintains physiological bone turnover. The diffusion of tumor cells in bone tissue breaks this process causing the disruption of bone integrity and serious skeletal complications [7-9]. Bone metastases from breast cancer are most often lytic, so that bone homeostasis is shifted toward bone resorption by osteoclasts.

Bisphosphonates are potent antiresorptive drugs in widespread use that are well suited to the treatment of metabolic bone disease. These drugs bind avidly to hydroxyapatite crystals at sites of active bone metabolism, achieving therapeutic concentrations. Bisphosphonates are released during bone resorption and are internalized by osteoclasts, leading to inhibition of bone resorption itself and induction of osteoclast apoptosis [10].

The use of drug treatments has a positive impact on the quality of life, inducing both a reduction of skeletal related events (SRE) and death risk in patients with bone metastases from breast cancer [11-13]. In particular, Zoledronic acid (Zol) is a potent third-generation nitrogen-containing bisphosphonate, and, in recent years, it has had widespread clinical use in patients with breast cancer [14]. Furthermore, many preclinical studies have demonstrated that Zol has both direct and indirect tumor activity, reducing proliferation and viability of tumor cell lines *in vitro*[15]. The direct action occurs in a dose and time dependent manner to inhibit proliferation and induce apoptosis in breast cancer cell lines [16]. The indirect action depends on the modification of bone microenvironment that is less hospitable for cancer cells’ growth. Furthermore, Zol is known to inhibit tumor cell adhesion and invasion [17,18] and its potential antiangiogenic activity has recently been discovered [19,20]. In animal models, a reduction in skeletal tumor burden and slower progression of bone lesions was observed after Zol treatment [21,22]. Recent clinical data in the adjuvant setting of breast cancer has also shown that Zol also increases disease-free survival [23,24].

However, one of the most important limitations of Zol which makes its direct anticancer effect difficult to demonstrate *in vivo* is its pharmacokinetics profile. After a 4-mg infusion the drug remains in the plasma circulation for 1–2 hours before localization to the bone, with a plasmatic peak of 1 μM. Studies on rats and dogs have shown that Zol levels rapidly decrease in plasma and non calcified tissue, whereas higher levels persist in bone and slowly diminish, with a half-life of about 240 days. Such results would seem to indicate that a part of Zol is reversibly taken up by the skeleton and that the disposition in blood and non calcified tissue is controlled by extensive uptake into and slow release from bone. The anticancer activity of this drug could be improved by increasing the availability of the drug in tissue outside the bone and by encapsulating it in liposome vehicles to lengthen its plasma half life. Other strategies could be to change the treatment schedule to low-dose protracted administration or to use synergistic combinations of drugs [25,26].

Several Zol dosing schedules have been proposed for the treatment of osteoporosis and bone metastases [27,28]. However, these schedules need to be optimized to maximize its antitumor effects [29,30]. The metronomic approach has already been studied, and, in particular, daily or repeated therapies with bisphosphonates have been reported to inhibit skeletal tumor growth in mouse models [21]. In cancer patients with bone metastases, repeated intermittent low-dose therapy with Zol has been shown to induce a decrease in VEGF levels in cancer patients.

The aim of this study is to compare the cytotoxic activity of repeated and non-repeated Zol treatment in 4 breast cancer cell lines that differ in their hormone receptors’ and HER2 status. We also investigated the molecular pathways involved in the antitumor activity of Zol.

## Materials and methods

### Cell culture

**Figure 1:**
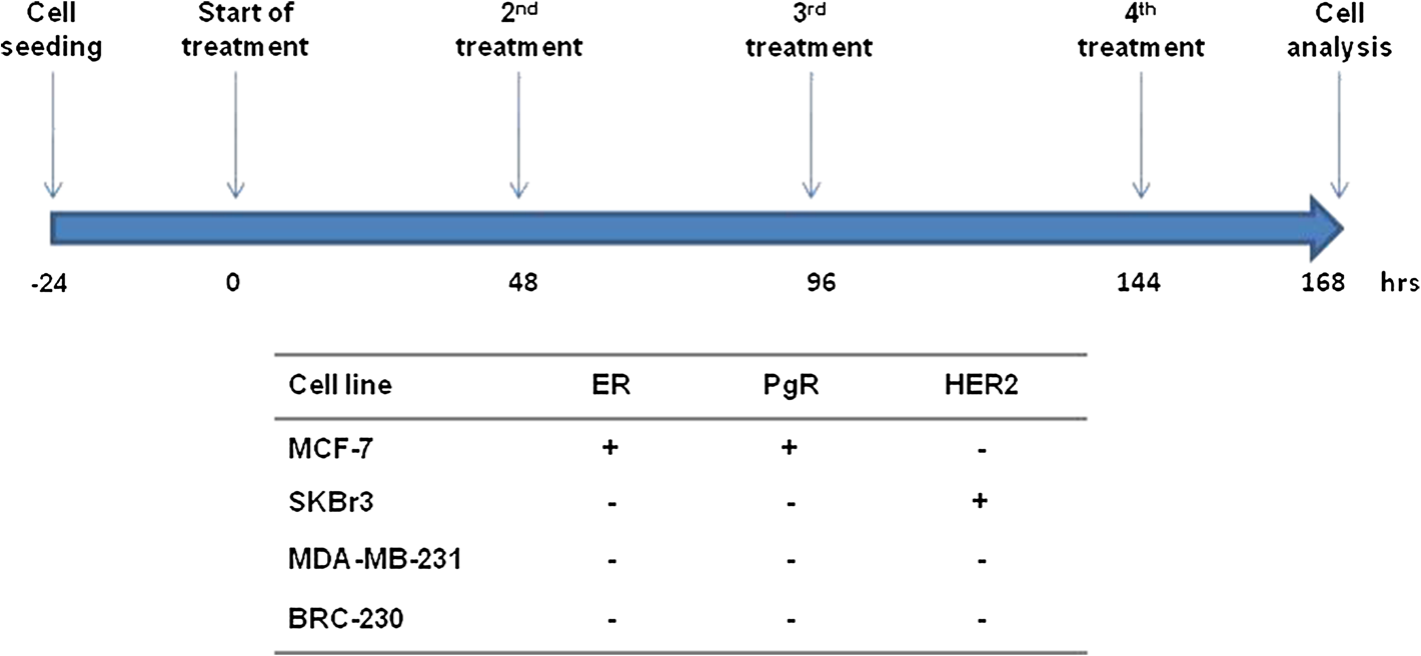
Treatment scheduling and biological characteristics of cell lines used.

The study was performed on four breast cancer cell lines, MCF-7, SKBr3, MDA-MB-231, obtained from the American Type Culture Collection (Rockville, MD), and BRC-230, established in our laboratory [31]. Hormone receptor and HER2 status are listed in Figure 1. The cell lines were maintained as a monolayer at 37°C and subcultured weekly. Culture medium was composed of 45% HAM F12 and 45% DMEM supplemented with 10% fetal calf serum, 1% insulin and 1% glutamine (Mascia Brunelli s.p.a., Milan, Italy). Cells were treated with Zol 24 hrs after seeding. Cells in the exponential growth phase were used for all experiments.

### Drugs

Zoledronic acid (Zometa®) (Zol), kindly provided by Novartis (Milan, Italy), was solubilized and stored at a concentration of 50 mM in sterile water at −20°C and diluted in medium before use. Cells were exposed to 12.5, 25 and 50 μM of Zol in chemosensitivity assay, and to 50 μM of Zol for apoptosis and western blot analysis.

### Chemosensitivity assay

Sulforhodamine B (SRB) assay was used according to the method of Skehan et al. [32]. Briefly, cells were trypsinized, counted and plated at a density of 3,000 cells/well in 96-well flat-bottomed microtiter plates (200 μl of cell suspension/well). In the chemosensitivity assay, experiments were run in octuplicate, and each experiment was repeated three times [33]. The optical density (OD) of cells was determined at a wavelength of 540 nm by a colorimetric plate reader. Growth inhibition and cytocidal effect of drugs were calculated according to the formula reported by Monks et al. [34]: [(OD_treated_ - OD_zero_)/(OD_control_ - OD_zero_)] × 100%, when OD_treated_ is ≥ OD_zero_. If OD_treated_ is above OD_zero_, treatment has induced a cytostatic effect, whereas if OD_treated_ is below OD_zero_, cell killing has occurred. The OD_zero_ depicts the cell number at the moment of drug addition, the OD_control_ reflects the cell number in untreated wells and the OD_treated_ reflects the cell number in treated wells on the day of the assay.

### Single drug exposure

In the chemosensitivity assay, cells were exposed to repeated (RS) and non-repeated schedules (NRS). In NRS experiments, cells were exposed for 168 hrs, while in the RS experiments, cells were exposed every 48 hrs to the same Zol concentration (Figure 1). All experiments were performed in triplicate and results were reported as the mean 50% inhibitory concentrations (IC50) of cell growth.

### Treatment of cells for apoptosis evaluation, western blot and pull-down assay

Cells were plated at a density of 10^6^ cells in a flask (75 cm^2^) and were treated 24 hrs after seeding with 50 μM of Zol according to the two schedules described above. For apoptosis analysis, cells were detached from the flasks by trypsin at the end of treatment, washed twice with PBS and stained according to the different methods specified below. For western-blot analysis, cells were detached from the flasks and were then lysed by shaking for 5 minutes in B-PER Mammalian Protein Extraction Reagent (Pierce, Rockford, IL). For pull-down analysis, post-treatment cells were stimulated by EGF 100 ng/ml for 10 minutes at 37°C (Miltenyi, Bologna Italy ) to evaluate Ras activity, and by Rho activator 1 (Cytoskeleton, Denver, CO) 1 U/ml for 30 min at 37°C to assess Rho activity. Cells were then washed once with PBS, lysed by cell lysis buffer (Cytoskeleton) and detached using a scraper. Protein concentration was assessed using BCA Protein Assay kit (Pierce).

### Wound scratch

Wound scratch assay was used to determine the migration of the four cell lines after Zol treatment (after 168 hrs). Cells were grown in flasks and the two treatments were performed. Twenty-four hours before stopping, a uniform cell-free area was created by scratching a confluent monolayer with a scraper. Wound closure was observed at the end of the experiments to determine cell line migration [35].

### Western blot

An equal quantity of proteins was denatured and separated on Criterion-HCL gel 12.5% Tris (Bio-Rad, Hemel Hempstead, UK) and electroblotted onto Immobilon-P Transfer Membrane (Millipore). The membrane was stained with Ponceau S (Sigma Aldrich, Milan, Italy) to verify equal amounts of sample loading and then incubated for 2 hrs at room temperature with T-PBS 5% non fat dry milk (Bio-Rad). The membrane was probed overnight at 4°C with the specific primary antibody, after which horseradish peroxidise-conjugated secondary antibody diluted 1:5,000 (Santa Cruz Biotechnology Inc, Santa Cruz, CA) was added. Bound antibodies were detected by Immun-Star Western C kit (Bio-Rad), using Chemidoc XRS Molecular Imager (Bio-Rad). The following primary antibodies were used: anti-p21 (monoclonal, 1:100) (BioOptica, Milan, Italy), anti-caspase 3 (polyclonal, 1:500), anti-caspase 9 (polyclonal, dilution 1:500), anti-bax (polyclonal, 1:1000), anti-pMAPK (polyclonal, 1:1000) (Cell Signalling Technology, Inc., Beverly, MA), anti-caspase 8 (monoclonal, 1:500) (Alexis Biochemicals, San Diego, CA), anti-RAS (polyclonal, 1:1000) (Stressgen, Exeter,UK), anti-Bcl-2 (monoclonal, 1:100) (Dako Corporation, Glostrup, Denmark), anti MCL-1 (monoclonal 1:100) (BD Pharmingen, San Jose, CA), anti rap1 (monoclonal 1:1000) (Abcam, Cambridge, UK) and anti-actin (polyclonal, 1:5000) (Sigma Aldrich), anti p-27 (monoclonal 1:2500) (BD Pharmingen, San Jose) and anti-MAPK (polyclonal 1:1000) (Cell Signaling Technology).

### Ras and Rho activity evaluation

The Ras/Rho Activation Assay Biochem kit (Cytoskeleton) was used according to the manufacturer’s instructions. Briefly, we performed a pull- down analysis of the RAF-RBD/GTP-Ras complex and GTP-RHO Rhotekin-RBD [36,37]. The amount of activated Ras was then determined by quantitative western blot using a Ras and Rho pan specific antibody. Band density was evaluated by Quantity one software.

### Apoptosis

For TUNEL assay, at the end of treatment cells were fixed in 1% paraformaldehyde in PBS on ice for 15 minutes, suspended in ice cold ethanol (70%) and stored overnight at −20°C. Cells were then washed twice in PBS and resuspended in PBS containing 0.1%

Triton X-100 for 5 minutes at 4°C. Thereafter, samples were incubated in 50 μl of solution containing TdT and FITC-conjugated dUTP deoxynucleotides 1:1 (Roche Diagnostics GmbH, Mannheim, Germany) in a humidified atmosphere for 90 minutes at 37°C in the dark, washed in PBS, counterstained with propidium iodide (2.5 μg/ml, MP Biomedicals, Verona, Italy) and RNAse (10 Kunits/ml, Sigma Aldrich) for 30 minutes at 4°C in the dark and analyzed by flow cytometry.

Flow cytometric analysis was performed using a FACSCanto flow cytometer (Becton Dickinson, San Diego, CA). Data acquisition and analysis were performed using FACSDiva software (Becton Dickinson). Samples were run in triplicate and 10,000 events were collected for each replica. Data were the average of three experiments, with errors under 5%.

### Cell cycle

After Zol treatment and the different washouts (168 hrs), cells were fixed in ethanol (70%), stained in a solution containing propidium iodide (10 mg/ml, MP Biomedicals, Verona, Italy), RNAse (10 kunits/ml, Sigma Aldrich) and NP40 (0.01%, Sigma Aldrich) overnight at 4°C in the dark, and analyzed by flow cytometry. Data were expressed as fractions of cells in the different cell cycle phases. A pulse-chase experiment was performed on MDA-MB-231 treated with RS to evaluate S Phase. MDA-MB-231 was used because it was the most sensitive cell line to Zol. Samples were taken at baseline, after 72 and 168 hrs’ treatment, and after a 48-hr washout.

### Bi-parametric BrdU-DNA content determination

BrdU (20 mM, Sigma Aldrich) was added to cell medium 15 minutes before the start of scheduled treatment. Cells were incubated using the previously reported drug concentrations or in control medium. At the end of each exposure time, cells were fixed in ice-cold ethanol (70%), stored overnight at −20°C, washed twice in PBS and incubated in HCl 2N for 25 min at room temperature. Samples were then washed with 4 ml of Na2B4O7 (0.1 M, pH8.5, Sigma Aldrich, Milan, Italy), incubated for 15 min at room temperature in PBS containing 0.5% Tween 20 (Biorad) and BSA 1% (Sigma Aldrich) and incubated with an anti-BrdU mouse antibody (NeoMarkers) (1/50 dilution in 0.5% Tween 20 and BSA 1%) for 1 hr at room temperature in the dark. Cells were washed in PBS and incubated with a FITC-conjugated anti-mouse immunoglobulin antibody (Dako Cytomation) (1/50 dilution in 0.5% Tween 20 and BSA 1%) for 1 h at room temperature in the dark. Before cytofluorimetric analysis, samples were washed with PBS and stained with propidium iodide 5 mg/ml (MP Biomedicals) and RNAse (MP Biomedicals) 1 mg/ml in PBS overnight at 4°C in the dark.

### Statistical analysis

Differences between dose response, apoptosis and schedules of treatments were determined using the Student's *t* test for unpaired observations. Statistical analyses were performed using the Statistical Package for Social Science (SPSS, version 17.0) and statistical significance was defined as *p* < 0.05. All *p* values were two-sided.

## Results

### Cytotoxic activity

**Figure 2:**
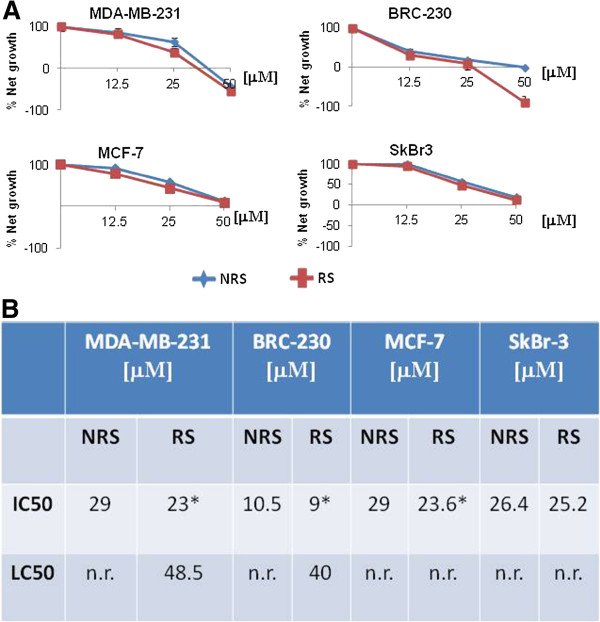
**Comparison between the two drug schedules, repeated (RS) and non-repeated (NRS).** Data represent the mean ± standard deviation (SD) of three independent experiments. Error bars represent the mean ± SD. **A**) Dose-effect curves of Zol in breast cancer cell lines. Standard deviation never exceeded 5%. **B**) IC50 and LC50 values in the 2 treatment schedules. Asterisks refer to values that differ significantly (*p* <0.05) with respect to control values. n.r., not reached.

Treatment cytotoxicity was assessed at scalar drug concentrations and IC50 value was calculated (Figure 2). The values of IC50 for RS were lower than those for NRS in all cell lines tested.

#### Triple negative cell lines

The NRS treatment induced, in MDA-MB-231 cells, a IC50 mean value of 29 μM compared to 23 μM for RS, with a decrease of 26% compared to standard treatment, (*p* =0.042) (Figure 2). BRC-230 cells were more sensitive to Zol for both schedules, and more specifically, the IC50 decrease was 14% greater with RS compared to NRS (*p* =0.003). Moreover, a cytocidal effect was observed with RS, inducing a LC50 of 49 μM and 40 μM in MDA-MB-231 and BRC-230, respectively.

#### MCF-7 and SkBr3 cell lines

NRS treatment induced IC50 values of 23.6 μM and 25.2 μM in MCF-7 and SKBr3, respectively, while the RS schedule resulted in IC50 values of 29.0 μM (MCF-7) and 26.4 μM (SKBr3) (Figure 2). Neither of the two treatment schedules induced a cytocidal effect. As the highest concentration produced the strongest effect in all cell lines, this was chosen for all subsequent experiments.

### Effect of zoledronic acid on the mevalonate pathway and proliferation markers

#### Triple negative cell lines

**Figure 3:**
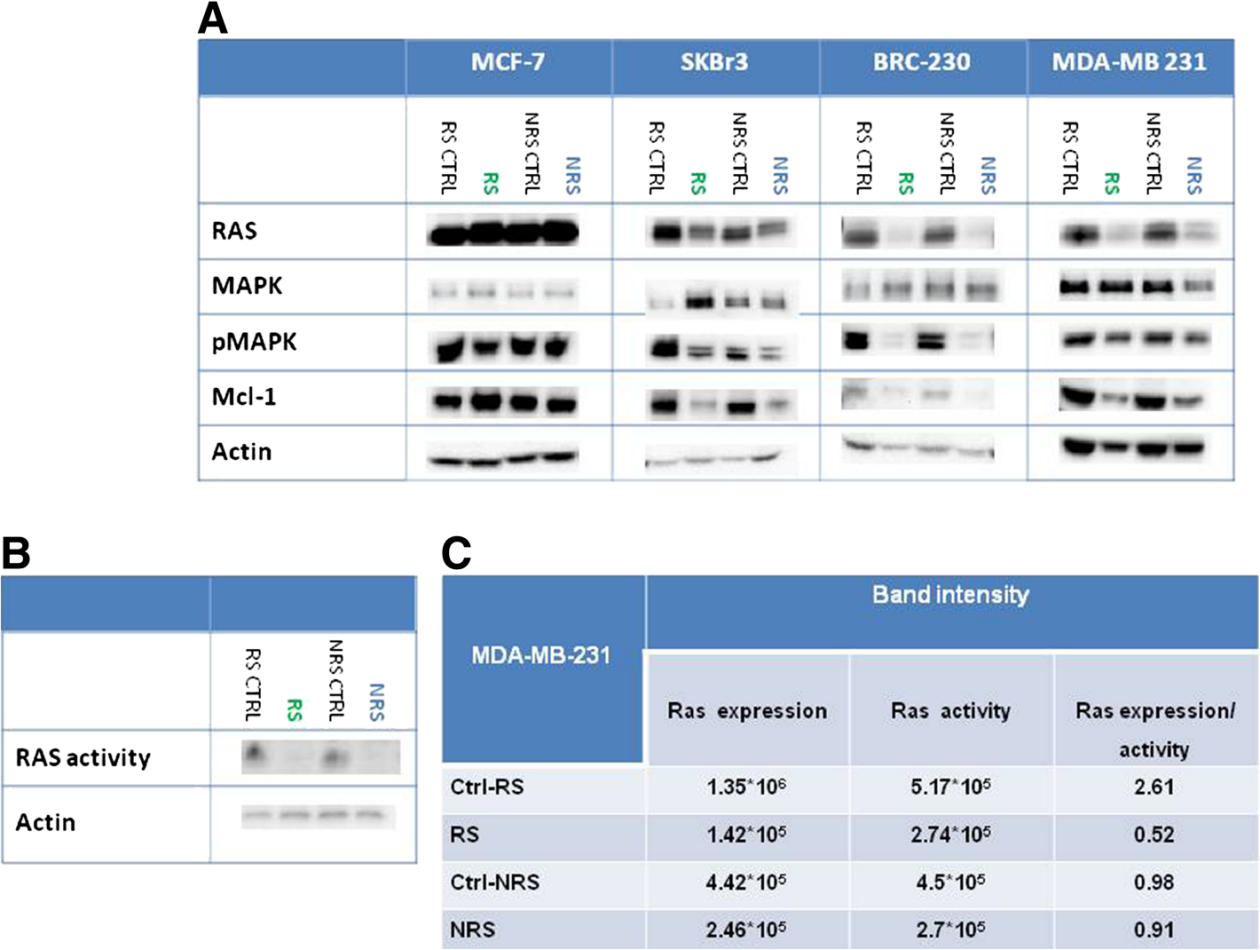
**Effect of Zol on the mevalonate pathway and on proliferation markers.A**) Western blot analyses. **B**) Ras activity evaluated by western blot. **C**) Band density quantifications by Quantity one software.

**Figure 4:**
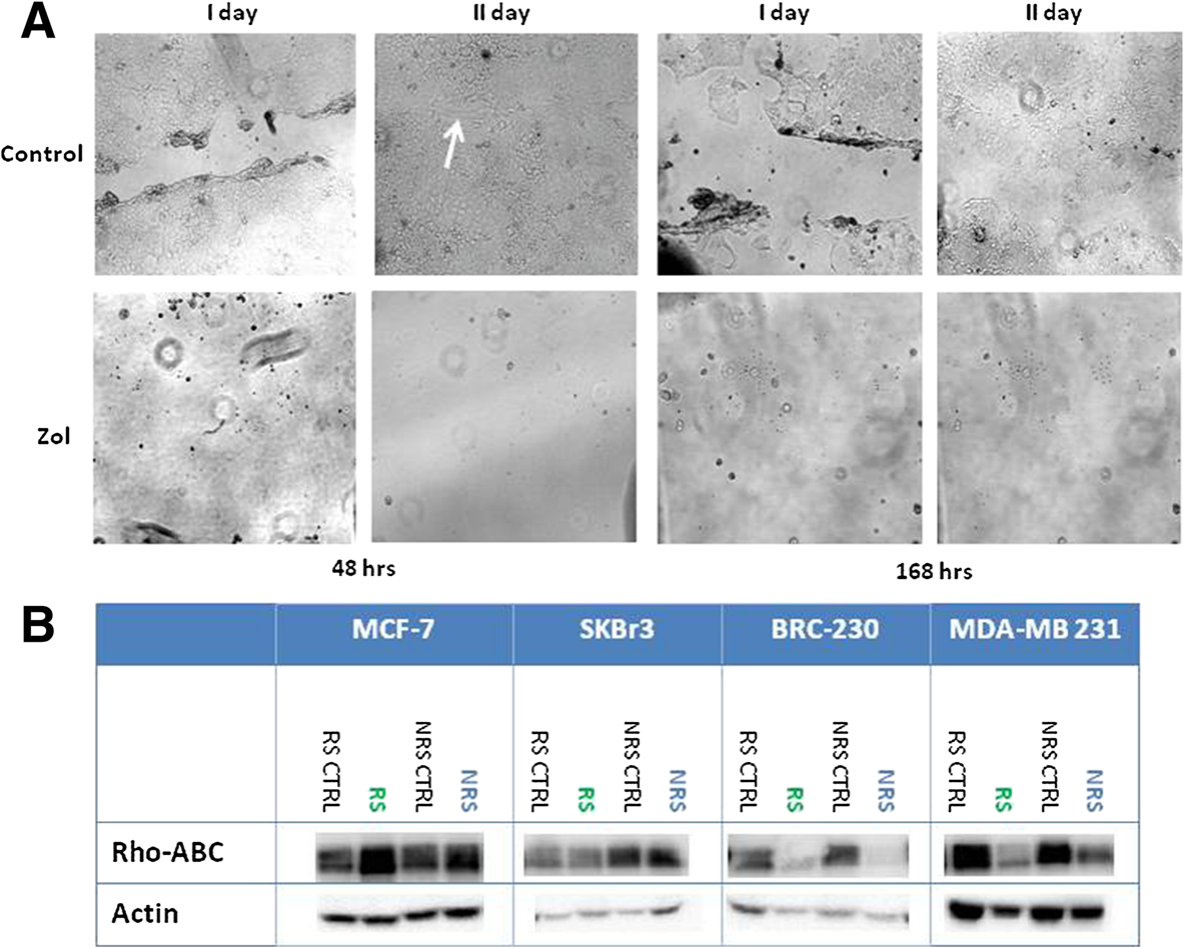
**Migration ability after treatment with Zol.A**) Wound scratch analysis to test the migration power of BRC-230 cells pre- and post-treatment. **B**) Evaluation of Rho expression by western blot analysis.

Both treatments induced a strong reduction in RAS expression in MDA-MB-231 and BRC-230 cells. There was no difference in MAPK levels after treatment in BRC-230 cell lines, whereas a strong decrease was observed after both treatments in MDA-MB-231 cells. Furthermore, a strong reduction of (Figure 3) pMAPK was observed in BRC-230 and, only slightly, in MDA-MB-231. Although both schedules inhibited the migration power of both cell lines, the reduction was more evident in BRC-230 (Figure 4). This result was confirmed by western blot analysis of RHO, which decreased after treatment.

**Figure 5:**
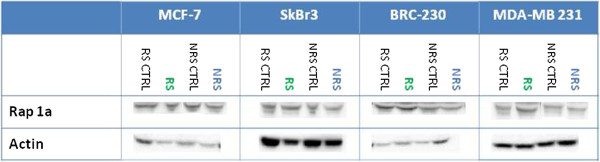
Evaluation of Rap1a levels by western blot analysis.

We evaluated Ras activity in MDA-MB-231 and observed a 50% decrease in its activity in both schedules. Ras expression levels decreased by about tenfold in cells exposed to RS and by about twofold in NRS-treated cells compared to control (Figure 5). Expressing this result as the ratio Ras expression/Ras activity in cells exposed to RS, we observed a difference between treated and non treated cells. No difference was found in Rho activity before or after treatment (data not shown).

#### MCF-7 and SkBr3 cell lines

In these two cell lines, the decrease in RAS and pMAPK was lower compared to that observed in triple-negative cells, and was more evident in SKBr3 cells (Figure 3). MAPK levels showed no change after treatment in MCF-7 cell lines, while a slight increase in expression was observed after treatment with RS in SKBr3. Both treatment schedules did not modify the migration power of either cell line. This result was also confirmed by the absence of modulation of RHO expression by western blot (Figure 4).

The differences observed in the cytotoxicity data and in the modulation of the mevalonate pathways cannot be attributed to a different uptake of Zol of the cell lines. In fact, no difference was detected in the accumulation of unprenylated Rap1A, a surrogate marker of Zol uptake (Figure 5).

### Apoptosis

#### Triple negative cell lines

**Figure 6:**
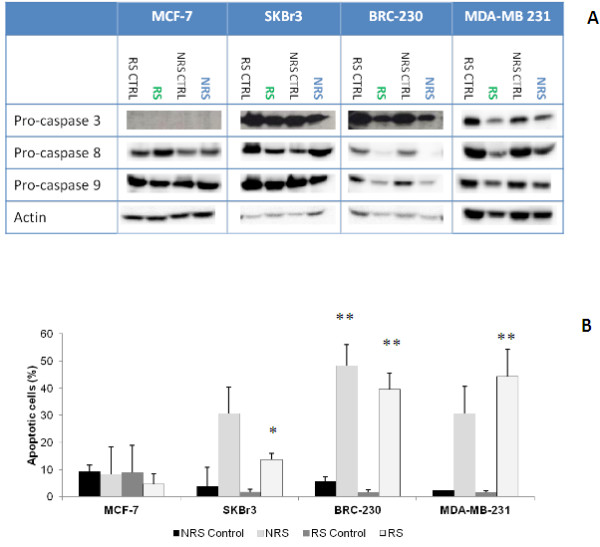
**Apoptotic cells evaluated by TUNEL assay.** Data represent the mean ± SD of three independent experiments. Error bars represent the mean ± SD. *p <0.05, ** *p* <0.01. **A**) Western blot data about modulation of apoptotic markers after the two treatment schedules in the 4 breast cancer cell lines. **B**) Percentage of cells in apoptosis. Data represent the mean ± SD of three independent experiments. *p <0.05, **p <0.01.

Zol induced apoptosis in both the triple negative cell lines used as experimental models (Figure 6). Both treatment schedules induced a significant percentage of apoptotic cells compared to the untreated control. However, MDA-MB-231 showed a higher percentage of apoptotic cells following RS compared to NRS treatment, without reaching statistical significance (44% compared to 30.6%). Conversely, BRC-230, showed a higher percentage of apoptotic cells after NSR treatment (48%) compared to RS (40%), without reaching statistical significance. Apoptosis was confirmed by western blot by a decrease in the levels of pro-caspase 3, 8 and 9 in both cell lines, without detection of the active forms. In MDA-MB-231, the levels of Bcl2 expression decreased after both treatments, whereas in BRC-230 the protein was not appreciably expressed (Figure 6). Furthermore, a decrease of mcl-1 expression was detected in both cell lines.

#### MCF-7 and SkBr3 cell lines

No apoptosis was observed in MCF-7, even if we detected the presence of debris, indicating early cell death. An almost complete disappearance of Bcl2 expression was also observed in MCF7 cells treated with RS. In SKBr3, the percentage of apoptotic cells was higher in treated cells following both treatment schedules compared to untreated control (not significant) (Figure 6). In addition, a strong reduction of MCL-1 was observed only in the SKBr3 cell line for both treatments. However, NRS treatment induced a higher percentage of apoptotic cells (31%) in this cell line compared to the RS treatment (14%).

### Cell cycle analysis

#### Triple negative cell lines

**Figure 7:**
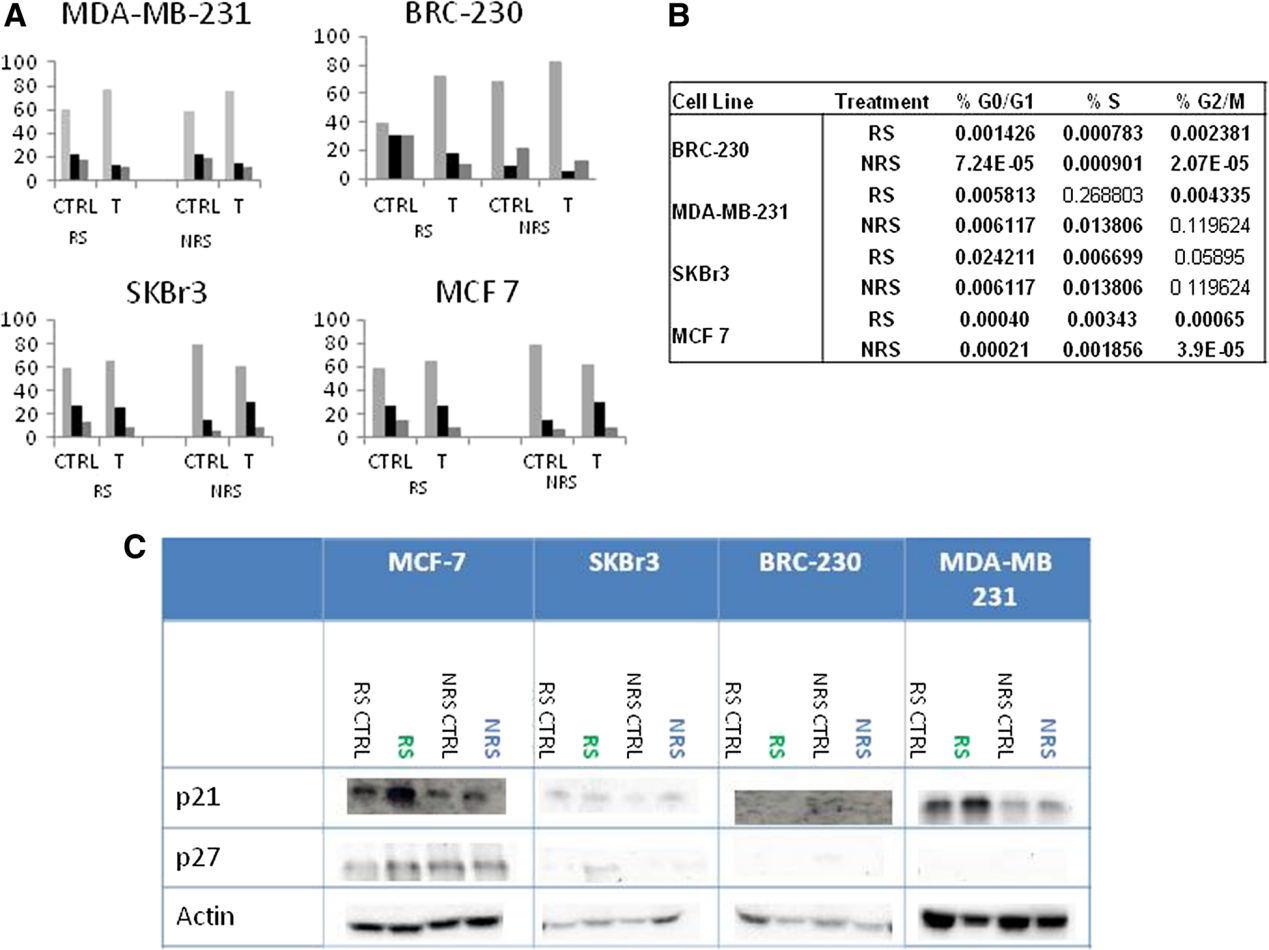
**Effect of Zol on the cell cycle.A**) Distribution of cells in the different cell cycle phases pre- and post-treatment. **B**) All *p* values were < 0.01 with the exception of S phase of BRC-230 cells exposed to NRS, G2/M phase of MDA-MB-231 cells exposed to NRS and G2/M of SkBr3 cells exposed to both treatments. **C**) Expression of p21 and p27 pre- and post-treatment in the different cell lines.

Both treatment schedules induced a significant increase of the percentage of cells in G0/G1 in all cell lines used (Figure 7) compared to untreated controls. The percentage of cells that accumulated in G0/G1 was 45.5% higher after RS with respect to control compared to NRS treatment (16.9%) in the BRC-230 cell line. This accumulation was also confirmed by the increase in p21 expression in RS in MDA-MB-231cells, whereas in BRC-230, the protein was not appreciably expressed. p27 expression was not evaluable in either of the two lines.

#### MCF-7 and SKBr3 cell lines

In SKBr3 cells, RS treatment induced an accumulation of cells in G0/G1 resulting in an increase of about 9% compared to untreated cells (*p* =0.005). Instead, NRS induced a cell accumulation in the S phase with a 50% increase in blocked cells compared to controls (*p* =0.01). Cell cycle perturbation was confirmed by an increase in p27 in both cell lines after Zol treatment.

**Figure 8:**
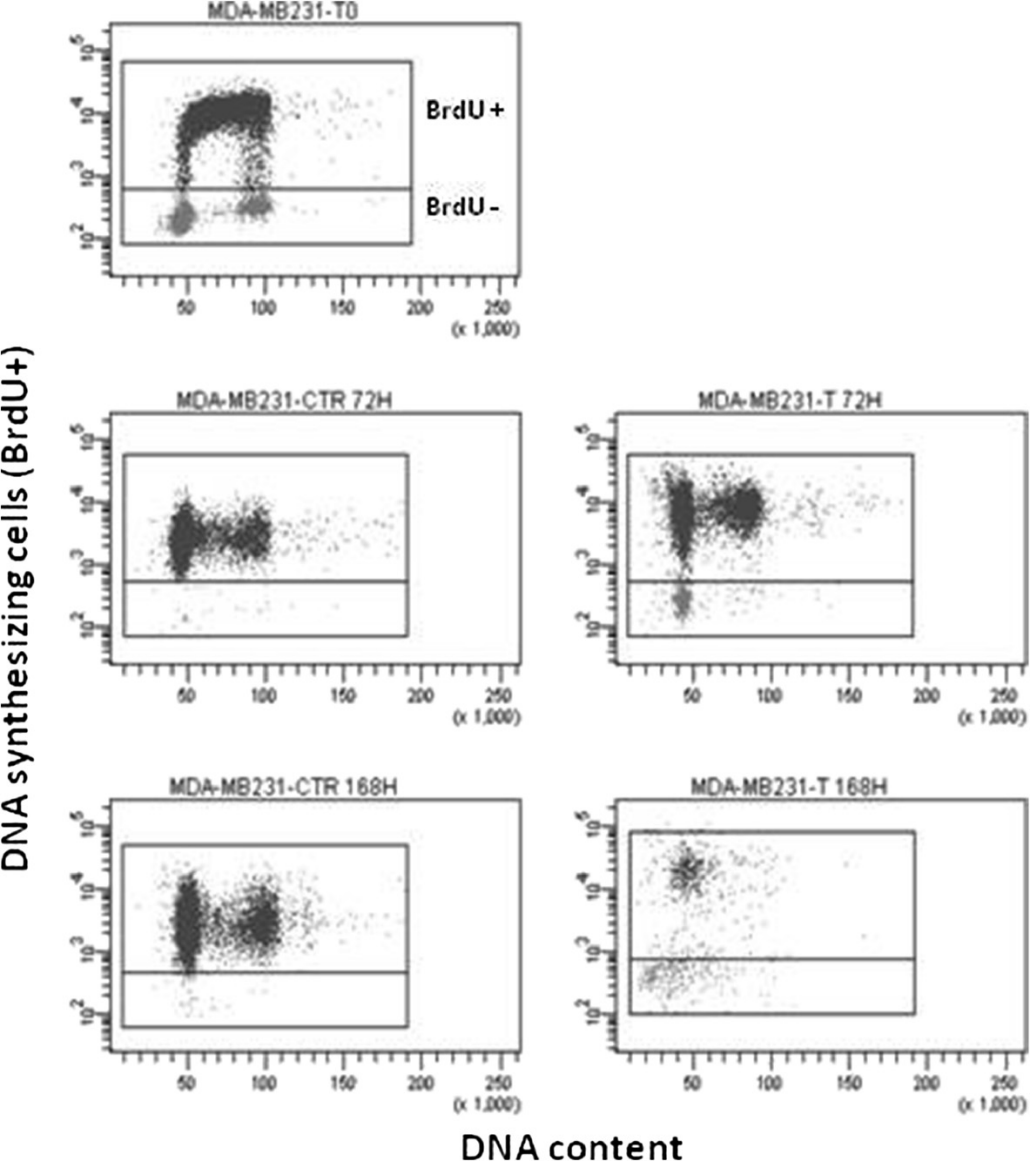
**Pulse-chase experiments on MDA-MB-231.** Experiments performed on cells treated with RS. Analysis of control cells at baseline, after 72 h and after 168 h (end of treatment), and of treated cells after 72 h and 168 h.

The pulse-chase experiment was performed on MDA-MB-231 exposed to RS. After 72 hrs and 168 hrs all untreated cells were BrdU-positive, indicating that every cell had entered S phase at least once and that there was a regular cell proliferation. Conversely, after the same times treated cells showed a fraction of BrdU-negative cells, confirming that Zol had arrested a certain percentage of cells in G0/1 phase. Of note, very few treated cells (BrdU-positive or -negative) entered or left S phase after 72 hrs, as also shown by the absence of clearly visible S and G2/M phases on the propidium iodide fluorescence axis (Figure 8). Forty-eight hrs after the end of treatment almost all cells were dead or in late-stage apoptosis and could thus no longer usable for S phase evaluation (data not shown).

## Discussion

In the present study, Zol induced cytostatic and cytocidal effects on breast cancer cell lines, in agreement with results from previous papers [38,39]. To mimic the bone microenvironment, concentrations of Zol used in our experiments (12.5, 25, 50 μM) were higher than the transient circulatory levels detected in patients. However, the concentrations used were in agreement with previously reported *in vitro* and *in vivo* data [15,40-43]. Moreover, it is well known that the pharmacokinetics and pharmakodinamic properties of Zol result in a rapid drug elimination by renal excretion and rapid uptake and accumulation within bone [44,45]. This accumulation has also been supported by a xenograft study which showed a high bisphosphonate concentration in bone compared to plasma [46]. For the reasons described above, we decided to use a higher concentration compared to that utilized in the clinical setting.

As expected, Zol exerted dose-dependent effects on cell proliferation in all cell lines following both treatment exposures. However, the repeated treatment induced a statistically significant modulation of cell proliferation and cytotoxic effect only in triple negative breast cancer cell lines. These data support results obtained in a preclinical model of bone metastasis induced in a triple negative cell line, showing that the antitumor effect of bisphosphonates increases when the drug is administered at low dose with a daily or weekly schedule [21], inducing a reduction of osteolyisis and growth of tumor in the bone.

Our results also confirm recent findings on the increased delivery of Zol to cancer cells both *in vitro* and *in vivo* through the use of liposomes or nanoparticles. It is hypothesized that the delivery of Zol by ‘stealth’ nanovectors mimics repeated administrations of Zol, and this would seem to be confirmed by the increased *in vitro* activity of the nanodevices encapsulating Zol [47-49].

Zol is known to block enzymes of the mevalonate pathway such as farnesyl pyrophosphate synthase, and/or geranylgeranyl pyrophosphate synthase [50]. This block causes a deficiency in isoprenoids which are essential for the post-translation lipid modification of signalling GTPases such as RHO and RAS [10,51,52]. To our knowledge, ours is the first Zol study on triple-negative lines to observe a modulation of RAS and RHO pathways; indeed, the decrease in RAS and pMAPK expression could explain the observed inhibition of cell proliferation. We also observed a decrease in RAS activity after treatment.

The wound scratch test showed a decrease of the migration power of triple-negative treated cells, most probably due to a decrease in RHO expression. The induction of the caspase pathway by Zol supports the hypothesis that apoptosis is linked to these pathways in triple-negative cell lines. Conversely, in HER2 overespressing (SKBr3) and positive hormone receptor cells (MCF-7), Zol induced only a cytostatic effect. In fact, a block (inhibition) of the RAS pathway was observed, with a reduction of pMAPK expression in SKBr3 cells, confirming the action of Zol to inhibit the mevalonate pathway.

There are conflicting literature data on breast cancer sensitivity to Zol, possibly due to the different HER2 and hormone receptors’ patterns of breast cancers. A study reported that MCF-7 and MDA-MB-231 cell lines were similarly sensitive to bisphosphonates [53]. Conversely, another study reported that clodronate reduced cell survival of MDA-MB-231, but not MCF-7 cells [54]. Hu et al. have characterized genetic alterations and oncogenic pathway in different breast cancers subtypes, both in tissue and in cell lines, and found that all mutations in *BRAF, KRAS* and *HRAS* were significantly associated with the triple negative subtype [55]. We hypothesized that triple-negative cell lines are more sensitive to Zol because the mevalonate pathway is blocked and the KRAS pathway is constitutively active. This hypothesis fits in with the MDA-MB-231 cell line profile, which harbors mutated *KRAS* and *BRAF*, while BRC-230 did not present any *BRAF*, *KRAS* and *HRAS* alterations (data not shown). However, BRC-230, presented a genetic amplification of EGFR and concomitant overexpression of the protein as observed in triple-negative breast cancers [56]. The hormone receptor (MCF-7) and HER2-positive (SKBr3) cell lines, not presenting any alterations in *BRAF, KRAS, NRAS, HRAS* or *EGFR*, appear to be less sensitive to both Zol schedules. A possible explanation could be the lack of caspase 3 in MCF-7 [41] and the overexpression of HER2 in SKBr3, which are involved in overcoming inhibition of the RAS pathway.

## Conclusions

Our work confirms the direct antitumor activity of Zol in human cell lines, as previously reported in *in vitro* and mouse models [21,22] and recently observed in patients in the Azure trial [57]. Furthermore, we highlighted an increase in the efficacy of Zol with repeated doses. In addition, the two triple-negative breast cancer cell lines were more sensitive to Zol than the other cell lines. These results indicate that it would be interesting to carry out further trials on animal models and, after successful completion, on patients. Finally, we performed an in-depth study of the mechanisms of action of Zol, observing that the KRAS/BRAF pathways are probably responsible for the sensitivity of the triple negative cell line. These data provide a sound rationale for using biologically targeted drugs for KRAS/BRAF/EGFR inhibition in combination with Zol. PARP inhibitors are another drug group that could potentially be used with Zol. Such hypotheses obviously need to be confirmed but are interesting in view of the limited treatment options available for patients with triple-negative breast cancer.

## Misc

Toni Ibrahim and Laura Mercatali contributed equally.

## Competing interests

The authors declare that they have no conflict of interests.

## Authors’ contributions

TI, LM and DA conceived the study and, together with AT and FF, participated in its design. LM and MZ performed part of the experimental work. ES carried out the chemosensitivity tests, CL the western blot analyses, SC cell cycle and apoptosis analyses, and PU the molecular genetic studies. TI, LM, AT and ES contributed to manuscript drafting. WZ and DA critically revised the manuscript for intellectual content. All authors approved the final version of the manuscript.
